# Pedicled Pronator Quadratus Transposition for Functional Opponensplasty: A Cadaveric Anatomical Study for Feasibility

**DOI:** 10.1177/15589447231153177

**Published:** 2023-02-14

**Authors:** Trey Cinclair, Lindsey Urquia, Austin Hembd, Shelby Lies

**Affiliations:** 1 The University of Texas Southwestern Medical Center, Dallas, USA

**Keywords:** hand, anatomy, carpal tunnel syndrome, nerve, diagnosis, surgery, specialty, nerve reconstruction, nerve injury, biomechanics, basic science, thumb

## Abstract

**Background:**

Techniques on opponensplasty for chronic carpal tunnel syndrome have been described previously. A novel pronator quadratus (PQ) transposition for chronic carpal tunnel syndrome is described. In addition, the relationship of the distal perforating branch of the radial artery to the surrounding tissue is detailed to optimize further use of the PQ flap for clinical applications.

**Methods:**

Ten cadaver hands underwent PQ dissection, and the perforating branch of the radial artery was identified. Measurements were taken from the radiocarpal joint and the radial styloid to the distal perforating branch. Finally, a proposed surgical technique of PQ transposition with proximal radius periosteum to the first metacarpophalangeal joint and anterior interosseous nerve transfer was performed.

**Results:**

The average distance of the perforating branch from the radiocarpal joint was 10 ± 1.05 mm, and the average distance from the radial styloid was 17.1 ± 1.6 mm. Pronator quadratus transposed with a layer of radius periosteum demonstrated anatomical feasibility.

**Conclusions:**

The distal perforating branch of the radial artery predictably perfuses the PQ muscle, which may be used in the future as a means of opponensplasty for chronic carpal tunnel syndrome.

## Introduction

Thenar muscle paresis can result from congenital etiology, chronic plexopathy with median nerve involvement, proper median nerve palsy, or, most commonly, advanced carpal tunnel syndrome. Prolonged compression of the median nerve alters neural blood flow dynamics, and eventual axonal death renders the thenar intrinsic muscles atrophied.^1^ Despite decompression with a carpal tunnel release and subsequent nerve regeneration, atrophy and normalized function are unlikely to return after marked chronicity.^1^ Thenar muscle impairment and the loss of palmar abduction, palmar flexion, and pronation components of thumb opposition may critically disable normal hand kinematics and serve as a barrier to activities of daily living.^2^ This study focuses on the treatment of thenar eminence atrophy secondary to advanced carpal tunnel.

Intervention with an opponensplasty may be indicated at the time of a carpal tunnel release or postoperatively depending on the chronicity and presentation of thenar impairment. One of the earlier techniques for opponensplasty was popularized by Camitz,^3^ who used the palmaris longus transfer for the insertion of abductor pollicis brevis (APB). Although indicated at the time of median nerve decompression for advanced carpal tunnel to assist with abduction, the motor is weak and likely too radial in vector, and insertion at the APB only restores palmar abduction.^4-6^ The Huber transfer uses the abductor digiti minimi, and benefits include restoring thenar bulk, but the vector lacks restoration of palmar abduction and commonly needs a free tendon graft to reach the thumb in adults.^5^ Therefore, if used, it is mostly performed in congenital thumb hypoplasia. The superficialis or extensor indicis proprius transfers improve on appropriate vector restoration but lack restoration of thenar bulk in marked thenar atrophy, which accompanies chronic carpal tunnel syndrome.^7^

Since the first description of the free pronator quadratus (PQ) muscle flap for soft tissue coverage of the forearm in 1984, case reports and small case series have described free functional transfer of the PQ muscle for loss of thenar muscle function. These reports are few and limited to traumatic injuries.^8-10^

In contrast, the primary aim of this study was to identify the anatomical reliability of a radial perforator. The secondary goal of this study was to demonstrate anatomical feasibility of a pedicled functional PQ flap in the prospective surgical management of thenar muscle dysfunction at the time of chronic carpal tunnel syndrome decompression. While previous studies have looked at the PQ free flap, this anatomical study looked at its feasibility as a pedicled flap. This technique, unlike the others described, could possibly both address functional opposition and improve the contour of atrophic thenar eminence.

## Methods

Ten fresh cadaver upper extremities (8 men and 2 women; mean age, 65.6 years; range, 60-75 years), amputated above the elbow, were acquired and prepared by means of the Willed Body Program. All specimens had no prior history of distal radius fracture.

**Figure 1.**
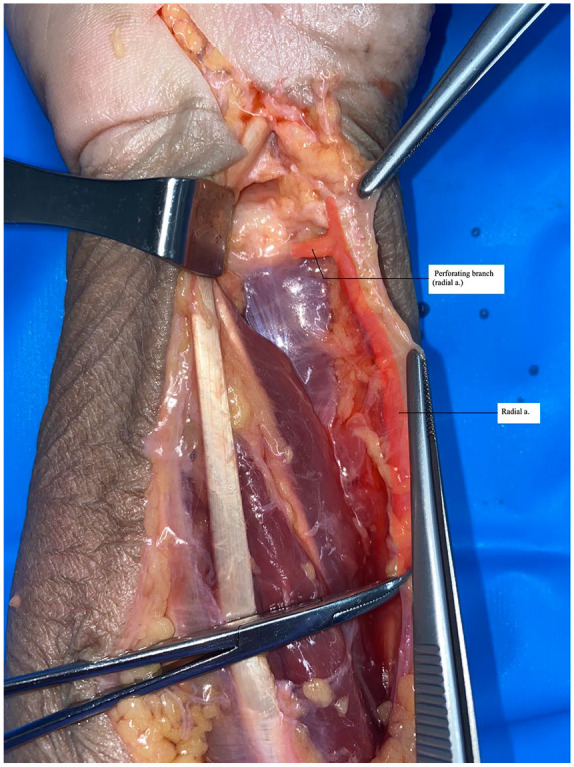
Hemostat clamping proximal radial artery with die injected: photograph of hemostat clamping proximal radial artery with color overlay highlighting the artery and a perforating branch.

**Figure 2.**
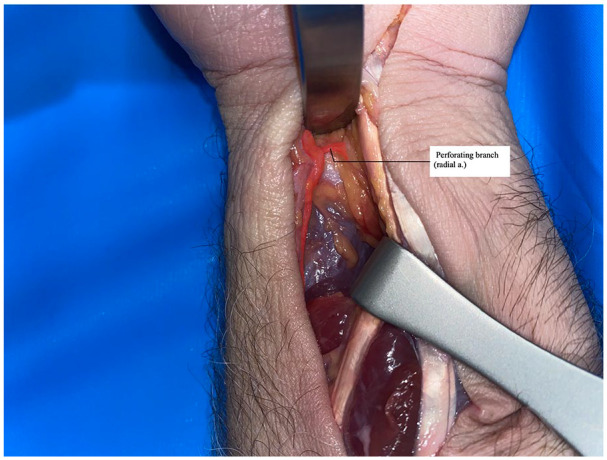
Perforating branch of radial artery exposed: photograph of perforating branch of radial artery dissection with color overlay highlighting the artery and a perforating branch.

**Figure 3.**
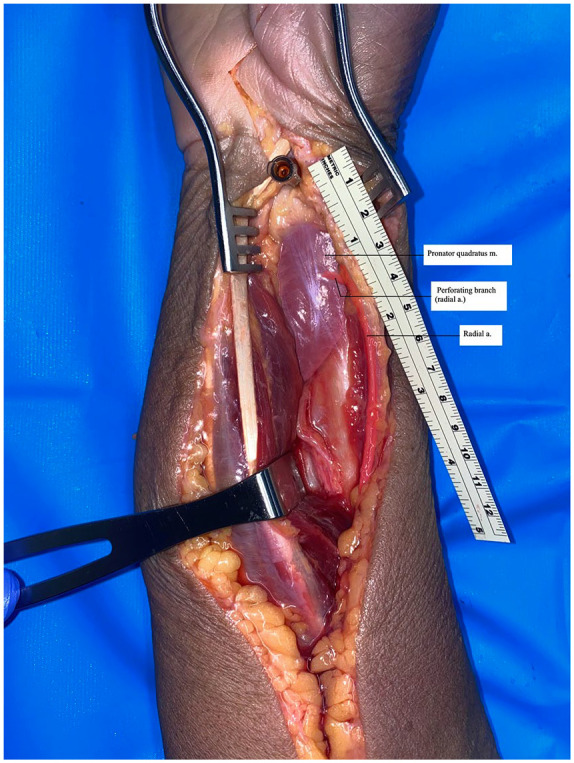
Measurement from the radial styloid to the distal perforator: photograph of the method used to measure the distance between the radial styloid and distal perforator with color overlay highlighting the artery and a perforating branch.

Ten PQ dissections were performed. An open carpal tunnel skin incision was made and extended to the forearm in line with a modified Henry approach; the flexor carpi radialis (FCR) and flexor pollicis longus (FPL) were retracted ulnarly and dissection was continued to the PQ muscle. The radial artery was then dissected and exposed to the level of the proximal one-third of the distal forearm. A hemostat was used to clamp the radial artery proximally, at which time a viscous red dye solution was injected distally until the specimen was perfused satisfactorily (Figure 1). Perforating branches of the radial artery were preserved and dissected free from surrounding tissues. Distal perforating branches to the PQ from the radial artery were identified and isolated (Figure 2). Two measurements were obtained: the distance from the radiocarpal joint to the most distal radial artery perforator, and the distance from the radial styloid process to the most distal perforator (Figure 3). Measurements were made to the nearest 0.1 mm. These measurements were used to calculate the mean distances and standard deviations.

**Figure 4.**
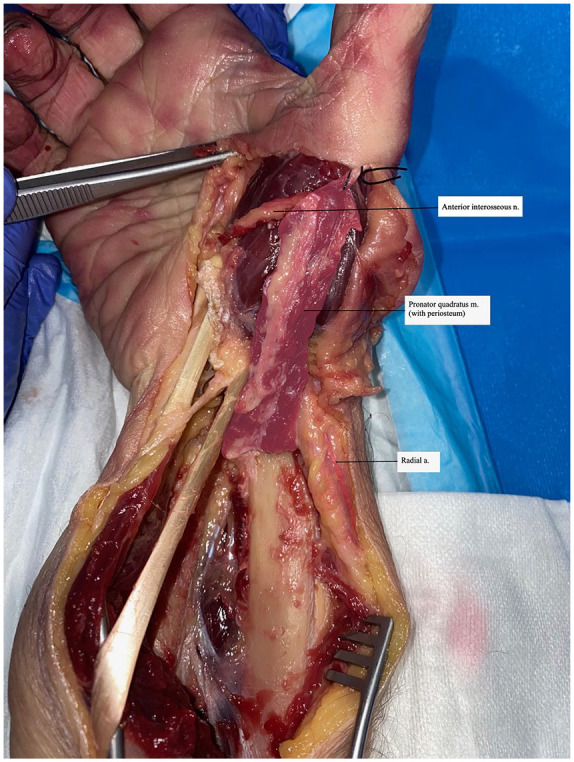
Pronator quadratus transposed with periosteum: photograph of pronator quadratus muscle with periosteum transposed to the thenar eminence with color overlay highlighting the muscle and artery.

After measurements were obtained, the anterior interosseous nerve (AIN) motor branch to the PQ and the accompanying anterior interosseous vessels were dissected proximally for maximal length. The PQ muscle with periosteum was first released from its proximal ulnar origin. It was then raised based on the distal perforating branch of the radial artery and transposed distally to the thenar eminence with the transected AIN (Figure 4). The anterior interosseous artery (AIA) and any additional proximal perforators from the radial artery were ligated to allow maximal movement of a distally based flap.

## Results

The average distance of the major distal perforator from the radiocarpal joint was 10 ± 1.0 mm. The average distance from the radial styloid process was 17.1 ± 1.6 mm (Table 1). Overall, a distal perforating branch consistently exited the radial artery within a 1- to 2-mm range to enter the PQ muscle.

**Table 1.** Results of Measurements on the Distal Perforating Branch of the Radial Artery.

Location of perforator	Average, mm	SD, mm
Distance from the radiocarpal joint	10	1
Distance from the radial styloid	17	2

A more proximal perforating branch of the radial artery was also found to contribute to PQ perfusion in all specimens at an average of 35 mm from the radiocarpal joint.

The PQ muscle, when raised with periosteum and pedicled on the distal perforating branch, was transposed to the level of the first metacarpophalangeal joint with adequate length to allow palmar abduction, palmar flexion, and pronation of the thumb (Figure 4).

### The Surgical Technique

The surgical approach for this procedure is through an extension of an open carpal tunnel incision extended to the forearm in line with a Henry approach, with or without an intervening skin bridge. Care needs to be taken in the subcutaneous plane at this area to identify and protect the palmar cutaneous branch of the median nerve. Once antebrachial fascia is open, the interval is developed between the FCR and FPL retracted ulnarly and the brachioradialis retracted radially. Identification of perforating arteries of the radial artery is carefully carried out, with a focus on the distal perforating branch to the PQ muscle. With the muscle completely exposed, a no. 15 blade is used to separate it from its ulnar origin along with a layer of radius periosteum. Care should be taken to raise the muscle and periosteum proximally to avoid damage to the AIN. Once the AIN was visualized, dissection is continued along the nerve proximally to afford maximal possible length until more proximal motor branches to the flexor digitorum profundus and FPL are encountered. Next, the PQ muscle is raised on its distal radial artery perforator, remaining attached only at its insertion into the distal radius. Around this attachment, the muscle is flipped over 180° distally and sutured into its insertional target at the first metacarpophalangeal joint. A proximal periosteal extension can assist with inset as needed. Finally, the AIN is reflected and coapted to the distal recurrent motor branch of the median nerve. The palmaris longus or FCR tendons or subsheaths can be potential pulleys to create a more palmar flexion vector if necessary, depending on anatomical variations and requirements.

## Discussion

Numerous technical variations of the opponensplasty exist, but the ability to provide adequate vectors of palmar flexion, abduction, and pronation while restoring thenar bulk in the setting of musculature atrophy in chronic carpal tunnel syndrome is limited with current techniques.^4-7^ This study of 10 cadaver specimens demonstrates the technical feasibility and pedicle reliability of the PQ muscle transposition opponensplasty, with the potential to restore the above listed components of function and contour.

While previous anatomical studies of the PQ have focused on muscle belly length and width, origin and insertion, and the course of the AIN and perforating branch of the AIA, both radial artery perfusion and potential for the muscle to reach the thumb metacarpophalangeal joint in a pedicled fashion have not previously been described.^11,12^ Coinciding with the results of Saint-Cyr et al,^13^ there were consistently 2 radial perforator arteries in our 10 cadavers. However, given the nature of our proposed transfer, it would not be feasible to keep the proximal perforator. Thus, we declined to study its anatomical significance at this time.

**Figure 5.**
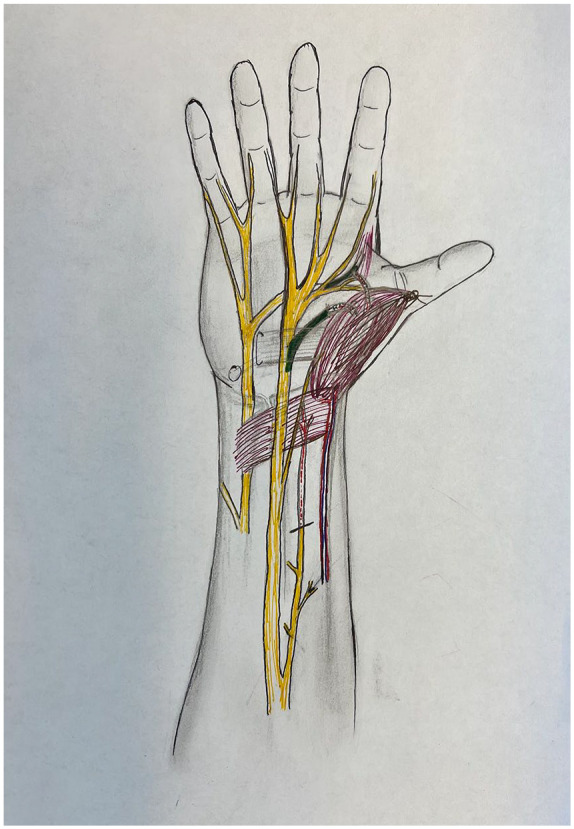
The nerves involved in pronator quadratus opponensplasty: image detailing the peripheral nerve anatomy of the hand. Recipient anterior interosseous nerve is highlighted in red. Donor recurrent median nerve and donor deep ulnar nerve are highlighted in green.

This technique may be limited to reconstruct opposition in a setting of chronic carpal tunnel and atrophic thenar muscle dysfunction, given the coaptation to the proximal recurrent motor branch of the median nerve. The AIN recipient nerve can be coapted to the recurrent motor branch if median nerve regeneration is expected within the 12- to 18-month timeframe or to the first dorsal interosseous muscle branch of the ulnar donor which is particularly more expeditious in the rare case of pronator compression (Figure 5). Decreasing the regeneration distance optimizes restored thenar muscle function. This would not be a functional coaptation in settings of more proximal median nerve palsy or disruption. A review by Rymer and Thomas^14^ reveals that the Camitz procedure yields good abduction but suffers in its ability to give pronation. Modified Camitz procedures have attempted to rectify this deficit with varying degrees of success. As this PQ muscle flap is pedicled from the distal radius in an oblique fashion, the vector crossing the radiocarpal joint provides true abduction out of the plane of the hand and the radioulnar direction of muscle fibers may more closely replicate the function of the opponens. Thus, this technique could offer better thumb abduction and pronation, given the position of the lever arm in this transposition while simultaneously restoring the thenar eminence. The degree of palmar flexion could be adjusted with subsheath or tendon pulleys with the FCR or FPL as necessary. The power of the PQ to pronate the forearm is more than sufficient to abduct thumb metacarpal assuming a normal carpometacarpal joint (CMCJ). Thus, severe CMCJ arthritis is contraindication for opponensplasty. This study shows that the distal perforating branch of the radial artery perfuses the PQ muscle with anatomical predictability and allows for adequate arc of mobilization around this pedicle to reach distal insertional targets. We also describe contribution from a more proximal perforating branch of the radial artery to be reliably present; this perforator necessitated sacrifice in our study of PQ transposition feasibility to achieve the necessary degree of motion.

Pedicled PQ muscle transposition to the thenar eminence is a feasible option for reconstruction of oppositional dysfunction in the setting of chronic carpal tunnel syndrome. The distal perforating branch of the radial artery perfuses the PQ muscle predictably to within 1 to 2 mm of margin, affording reliability and technical feasibility based on this cadaver study.
